# Survival, prognostic factors, hospitalization time and clinical performance status after first cerebral relapse or progression in 54 patients with primary CNS lymphoma not eligible for high dose chemotherapy: a retrospective analysis

**DOI:** 10.1186/s42466-023-00234-y

**Published:** 2023-02-23

**Authors:** Sabine Seidel, Thomas Kowalski, Verena Nilius-Eliliwi, Roland Schroers, Uwe Schlegel

**Affiliations:** 1 Department of Neurology, University Hospital Knappschaftskrankenhaus, Ruhr University Bochum, In der Schornau 23-25, 44892 Bochum, Germany; 2 Department of Hematology and Oncology, University Hospital Knappschaftskrankenhaus, Ruhr University Bochum, In der Schornau 23-25, 44892 Bochum, Germany

**Keywords:** r/r PCNSL, Primary CNS lymphoma, Salvage treatment, Relapse, High-dose methotrexate, Temozolomide, Whole brain radiotherapy

## Abstract

**Background:**

Treatment of relapsed or refractory primary CNS lymphoma (r/r PCNSL) is difficult, particularly in patients not eligible for high dose chemotherapy with autologous stem cell transplantation (HDC-ASCT). No standard treatment has been defined for these patients yet.

**Methods:**

We retrospectively analyzed survival, prognostic factors, hospitalization time and Karnofsky performance score (KPS) before and after treatment in 54 r/r PCNSL patients with isolated cerebral relapse or progression (n = 23 refractory, n = 31 relapsed) not eligible for HDC-ASCT, who received heterogenous salvage treatments.

**Results:**

Treatments were temozolomide (+ rituximab) (n = 21), high dose methotrexate (HD-MTX)-based therapy (n = 11), whole brain radiotherapy (WBRT)/focal radiotherapy (n = 11), other systemic treatments (n = 2) and best supportive care (BSC, n = 9). Median progression free survival (PFS) and overall survival (OS) were 2.6 months (95% CI 1.0–4.2 months) and 4.8 months (95% CI 3.3–6.3 months), respectively. Eight patients survived for ≥ 3 years (13.1%, n = 3 received temozolomide, n = 3 WBRT, n = 2 HD-MTX-based treatment). Application of any salvage treatment (vs. BSC), younger age at relapse and asymptomatic (vs. symptomatic) relapse were positive prognostic factors. No significant differences in OS were found for the different salvage treatments. Median hospitalization time for treatment was 15/13 days for temozolomide (+ rituximab)/radiotherapy compared to 55 days for HD-MTX-based therapy. Median KPS in assessable patients (n = 41) was 60 (range 30–100) before treatment and 50 (range 20–90) after treatment. In patients with response to treatment (n = 16) KPS improved from 60 (range 40–90) before treatment to 70 (range 50–90) after treatment, while patients with PD (n = 25) deteriorated from 60 (range 30–100) to 40 (range 20–70).

**Conclusion:**

Survival for this cohort of r/r PCNSL patients with isolated cerebral relapse or progression was poor. Considering long hospital stays associated with HD-MTX-based chemotherapy and neurotoxicity associated with WBRT, temozolomide might be worth considering with a chance of prolonged survival and avoidance of long hospitalization. Novel therapeutic agents are urgently needed to improve survival in r/r PCNSL patients.

## Background

The prognosis of primary central nervous system lymphoma (PCNSL) has improved significantly during the last decades due to the implementation of high-dose methotrexate (HD-MTX)-based chemotherapy as first line treatment [1]. However, about one third of patients have refractory disease during first line treatment [2, 3] and about half of the patients with complete response (CR) after first line eventually relapse [3].

No standard treatment has been defined for relapsed or refractory primary central nervous system lymphoma (r/r PCNSL) yet [4]. The only treatment associated with durable responses in a fraction of patients is high-dose chemotherapy with autologous stem cell transplantation (HDC-ASCT) [5–8]. However, the majority of PCNSL patients at relapse are not able to receive HDC-ASCT [2]. To date there is no standardized scoring system for the evaluation of eligibility for HDC-ASCT and the decision is mainly based on the treating physicians` overall assessment. Factors considered for the decision are age, clinical status evaluated by the Karnofsky performance score (KPS) or Eastern Cooperative Oncology Group (ECOG) score, comorbidities (a commonly used scoring system is the Charlson`s comorbidity index [9]) as well as renal, lung, bone marrow and cardiac function. In patients with relapse of PCNSL an additional factor considered for the decision for or against HDC-ASCT is the tolerance of and the toxicities associated to initial treatment.

Therapeutic options for patients with cerebral relapse not able to receive HDC-ASCT comprise HD-MTX-rechallenge, whole brain radiotherapy (WBRT), a variety of Non-HD-MTX-based chemotherapy regimens, or novel systemic drugs such as immune checkpoint inhibitors or targeted therapies preferably within clinical trials. HD-MTX-rechallenge in relapsed patients is a valuable option for salvage treatment in patients with long remissions after HD-MTX-based first line treatment; two retrospective studies reported median overall survival (OS) times of 41 [10] and 61.9 [11] months. For WBRT median OS of 10.9 [12] and 16 [13] months have been reported in two retrospective studies, however, neurocognitive decline in a relevant fraction of patients has been reported in both series. Many small retrospective and prospective studies on different non-myeloablative, non-HD-MTX-based chemotherapy regimen have been published. Regarding only studies with inclusion of more than 20 patients, median OS of 3.9–11.2 months have been reported with rituximab + pemetrexed [14], temozolomide (TMZ) [15], topotecan [16], ifosfamide and etoposide in combination with carboplatine [17], or rituximab [18], or rituximab + thiotepa [19]. More recently, an increasing number of studies on targeted therapies has been published. Studies with more than 20 patients reported median OS of 3.7–19.2 months with ibrutinib monotherapy [20], lenalidomide + rituximab [21], pemetrexed + lenalidomide [22], pomalidomide + dexamethasone [23] and temsirolimus [24].

In this retrospective study we analyzed survival and prognostic factors in 54 patients, refractory to first line treatment or at first cerebral relapse of PCNSL and not eligible for HDC-ASCT, who had received heterogenous salvage treatments at our institution. Considering the generally unfavorable prognosis, several therapeutic aspects beyond mere survival are important for these patients. Therefore, we retrospectively reviewed several factors associated with therapy, that might have an impact on quality of life and an influence on the benefit associated with treatment as treatment duration, time of hospitalization and the Karnofsky Performance Score (KPS) before and after treatment.

## Patients and methods

All consecutive HIV negative patients that presented with isolated cerebral manifestation of r/r PCNSL between January 2006 and June 2022 not eligible for HDC-ASCT, as defined below, were included into this analysis. All patients had been treated with HD-MTX-based polychemotherapy as first line treatment at our hospital. Patients were included in case of radiologically confirmed relapse, progression of PCNSL during or immediately after completion of first line treatment, or in case of partial response only after completion of initial treatment. Whole body fluorodeoxyglucose (FDG)-positron emission tomography (PET)/computer tomography (CT) (or CT of neck, chest and abdomen and ultrasound of the testes) as well as ophthalmological and cerebrospinal fluid (CSF) examinations were carried out for staging at relapse. Baseline examinations to determine eligibility for HDC-ASCT were evaluation of clinical condition, of comorbidities (using the Charlson Comorbiditiy Index [CCI] [9]) and of renal, bone marrow, cardiac and pulmonary function. All treatment decisions were made in consensus among hematooncologists and neurologists. Treatment of relapse was not standardized and determined by duration of remission after HD-MTX at first-line (in order to opt for HD-MTX rechallenge), on patients` comorbidities and possible preferences, and on hospital treatment guidelines established at time of salvage therapy. Primary CNS Lymphoma Collaborative Group (IPCG) criteria were used for evaluation of response [25]. After completion of salvage treatment, regular clinical and ophthalmological examinations as well as cerebral magnetic resonance imaging (cMRI) were performed as part of a routine follow-up program with intervals based on recommendation for first line treatment (quarterly for two years after completion of therapy, every six months for three more years and annually afterwards [4, 26]). If leptomeningeal involvement was present at primary progression/first relapse, CSF was also examined at those regular follow-ups. Clinical, laboratory and imaging data, treatment duration, hospitalization time, discharge destination and clinical status before and after treatment were obtained from patient charts and the archive of the hospital. Clinical status was evaluated between zero and four weeks after treatment termination or completion. Censoring date was June 30, 2022. The Ethics Committee of the University of Bochum, Faculty of Medicine approved the study.

Median follow-up was defined as median observation time after diagnosis of progression or relapse. Treatment duration was defined as the time from first day of salvage treatment until discontinuation of treatment for any reason (including death) or completion of treatment. Hospitalization time was defined as the time spent hospitalized during treatment for first relapse/progression for any reason. Progression free survival (PFS) was defined as the time from diagnosis of relapse to next relapse or progression, death of any cause (if progression was not determined) or last date of follow-up. OS was calculated from diagnosis of relapse to death of any cause or last date of follow-up. OS and PFS were estimated by the Kaplan–Meier method. To compare OS and PFS between groups log-rank tests were used. Multivariate analysis was performed using the Cox proportional hazard regression model. The variables for multivariate analysis were determined based on previously published series on treatment of r/r PCNSL [2, 27]. The level of significance was 0.05 (two-sided). Analyses were conducted using SPSS (version 23).

## Results

### Patient characteristics

A total of 120 patients presented at our hospital with relapse after or refractory disease to HD-MTX-based polychemotherapy as initial treatment for PCNSL between 2006 and 2022. Of those, 59 were deemed eligible for HDC-ASCT; clinical results for those patients have been reported elsewhere [7]. Sixty-one patients were not eligible for HDC-ASCT. Five of these patients had isolated systemic relapses and two patients had isolated ocular relapse. Isolated cerebral relapse or progression was observed in 54 patients (n = 23 refractory, n = 31 relapse) and these patients were included into the analysis. Median follow-up was 4.7 months (range 0.1–154.1). Median age at diagnosis of progression or relapse was 73.5 years (range 26–85) and median KPS was 60 (range 30–100). Two patients had developed other malignancies within five years prior to diagnosis of PCNSL (n = 1 breast cancer treated by resection, radiotherapy and antihormonal treatment; n = 1 prostate cancer treated by resection and antihormonal treatment). Two patients had received immunosuppressive medication (n = 1 continuous steroid medication for alveolitis, n = 1 low dose MTX for polyarthritis). Two patients (3.7%) were lost to follow-up.

Responses to first line treatments had been complete remission (CR) or complete remission unconfirmed (CRu) in 31 patients (57.4%), partial remission (PR) in four patients (7.4%), and progressive disease (PD) in 19 patients (35.2%), respectively. Median time from last day of prior treatment to first relapse was 13.7 months (range 2.4–83.5) in patients, who initially had CR/CRu after first line treatment (n = 31). Details on patient characteristics are given in Table 1.

**Table 1:** Patient characteristics at first relapse/progression (n = 54)

	No. of patients
Age, years	
Median (range)	73.5 (26–85)
≥ 65	46 (85.2%)
< 65	8 (14.8%)
KPS	
Median (range)	60 (30–100)
≥ 70	15 (27.8%)
< 70	39 (72.2%)
Gender	
Male	25 (46.3%)
Female	29 (53.7%)
Cognitive impairment at diagnosis of relapse ^a^	
Yes	30 (55.6%)
No	24 (44.4%)
Hemiparesis at diagnosis of relapse	
Yes	12 (22.2%)
No	42 (77.8%)
Seizures at diagnosis of relapse	
Yes	8 (14.8%)
No	46 (85.2%)
Steroid use at diagnosis of relapse	
Yes	28 (51.9%), median dose 12 mg (range 2–24 mg)
No	26 (48.1%)
Involvement of DBS	
Yes	38 (70.4%)
No	16 (29.6%)
Distribution of lesions in cMRI	
Unifocal	22 (40.7%)
Multifocal	30 (55.6%)
Diffuse leptomeningeal	2 (3.7%)
CSF cytology	
Positive	4 (7.4%)
Negative	13 (24.1%)
Not done	37 (68.5%)
Charlson Comorbidity Index	
Score 0	35 (64.8%)
Score ≥ 1	19 (35.2%)
Status before salvage treatment	
Relapse	31 (57.4%)
Refractory	23 (42.6%)
Relapse/progression occurred in	
2006–2009	13 (24.1%)
2010–2015	22 (40.7%)
2016–2022	19 (35.2%)

^a^Patients with cognitive impairment documented in neurological examination at relapse, no standardized neurocognitive or neuropsychological assessment was performed

*Abbreviations:* CSF = cerebrospinal fluid, DBS = deep brain structures, KPS = Karnofsky performance score, MRI = magnet resonance imaging

### Treatment of relapse

**Fig. 1:**
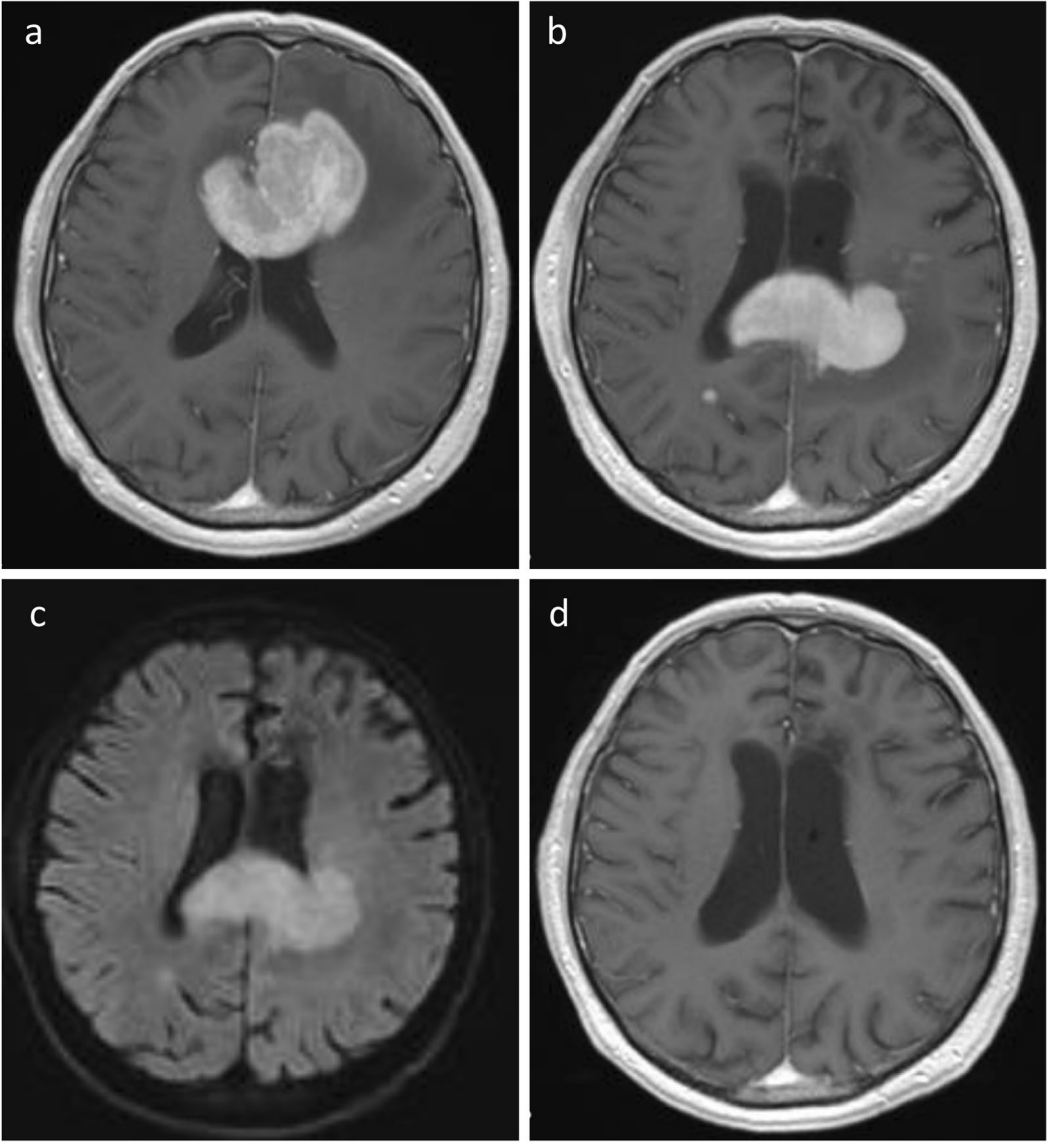
cMRI imaging of a 63 years old patient at **a** initial diagnosis (T1 weighted imaging with contrast medium), **b** at relapse (T1 weighted imaging with contrast medium), **c** also at relapse (diffusion weighted imaging) and **d** at remission after HD-MTX based retreatment plus focal radiotherapy (T1 weighted imaging with contrast medium)

Of 54 patients, 21 patients (38.9%) received TMZ (n = 15 TMZ monotherapy, n = 6 TMZ + rituximab) as salvage treatment. Eleven patients (20.4%) received a rechallenge with HD-MTX-based treatment (n = 8 HD-MTX + TMZ, n = 2 HD-MTX monotherapy, n = 1 HD-MTX + ifosfamide). In this group one patient received WBRT at PR after four treatment cycles because of infectious complications and one patient with a single lesion at relapse received consolidating focal radiotherapy at PR after HD-MTX-based treatment (cMRI imaging of this patient is displayed in Fig. 1). Eleven patients (20.4%) received salvage radiotherapy (n = 10 WBRT, n = 1 local radiotherapy). One of these patients refused to continue treatment after 14 days. Two patients (3.7%) received other systemic treatments (n = 1 a mammalian target of rapamycin [mTOR] inhibitor [temsirolimus] within a clinical study [24], n = 1 rituximab monotherapy because of concomitant spondylodiscitis as contraindication for more intensive treatment). Nine patients (16.7%) received best supportive care (BSC) only.

Eleven patients in this cohort were < 65 years of age at diagnosis of relapse or progression and were still not considered eligible for HDC-ASCT. In seven of these patients severe, mainly infectious and hematologic complications occurred during initial treatment and these patients were therefore considered unable to tolerate HDC-ASCT. Four patients had comorbidities leading to ineligibility for HDC-ASCT.

### Response to treatment and survival

Nine of 54 patients received BSC only. Of the 45 patients who received salvage treatment, response to treatment was CR/CRu in 14 patients (31.1%), PR in two (4.4%), PD in 25 (55.6%) and treatment related death in four patients (8.9%). Of those patients, who died during treatment, two patients developed pneumonia associated sepsis (n = 1 treated with rituximab + TMZ, n = 1 treated with rituximab + HD-MTX + TMZ). One patient had a subarachnoid hemorrhage from an anterior communicating artery aneurysm, deteriorated clinically, and died due to pneumonia. One patient died for unknown reasons before assessment of treatment response. At censoring date, 48 of 54 patients had died. Causes of death were: progression of PCNSL (n = 35), unknown (n = 5), early death during treatment of first relapse (n = 4), complications related to WBRT-induced encephalopathy (i.e., severe dementia and death from pneumonia associated with immobilization, n = 1), and sepsis due to pneumonia 3.5 months after WBRT as second salvage treatment (n = 3).

**Fig. 2:**
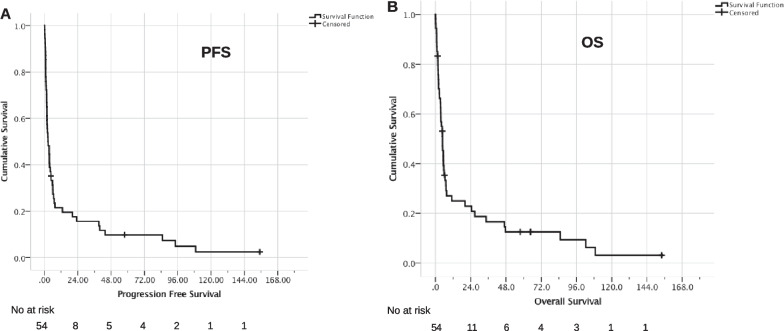
Progression free survival (**a**) and overall survival (**b**) in patients with cerebral relapse/progression (n = 54)

Median PFS was 2.6 months (95% CI 1.0–4.2, Fig. 2A). 1-year PFS was 21.5%, 2-year PFS 15.6%, and 5-year PFS 9.8%. Median OS after cerebral relapse/progression was 4.8 months (95% CI 3.3–6.3, Fig. 2B). 1-year OS was 25.0%, 2-year OS 22.9%, and 5-year OS 12.5%.

### Prognostic factors

Patients who received any salvage treatment had a significantly longer median OS (5.3 months [95% CI 4.2–6.4]) as compared to patients with BSC only (0.8 months [95% CI 0.5–1.0], *p* < 0.001).

Patients < 75 years had a significantly longer median OS (7.0 months [95% CI 4.2–9.8]) than patients ≥ 75 years (2.7 months [95% CI 0.6–4.7], *p* = 0.007). In patients < 75 years, 1-year OS, 2-year OS, and 5-year OS rates were 34.1%, 29.9%, and 21.3%, respectively. In patients ≥ 75 years 1-year OS, 2-year OS, and 5-year OS rates were 15.4%, 15.4%, and 3.8%. All 26 patients ≥ 75 years had died at censoring date (n = 7 had received BSC only). Four patients ≥ 75 years had documented survival of ≥ 12 months (n = 2 treated with WBRT, n = 1 TMZ, n = 1 HD-MTX + TMZ).

No significant difference in OS in univariate analysis was found for the following factors: KPS, gender, CCI, involvement of deep brain structures at relapse, status before salvage treatment (refractory vs. relapsed), symptoms at relapse (symptomatic vs. asymptomatic), time in remission before relapse, treatment with radiotherapy (vs. any other treatment including BSC), HD-MTX-based treatment (vs. any other treatment including BSC), TMZ monotherapy/TMZ + rituximab (vs. any other treatment including BSC).

In multivariate analysis younger age (*p* = 0.003) and asymptomatic relapse/progression (*p* = 0.040) were positive prognosticators (see Table 2).

**Table 2:** Multivariate analysis of prognostic factors for OS after cerebral relapse (n = 54)

Variables	HR	95% CI	p-value
Age (continuous)	1.071	1.024–1.121	**0.003**
KPS (continuous)	0.992	0.965–1.020	0.571
Gender (male vs. female)	0.588	0.277–1.246	0.166
Charlson comorbidity score 0 vs. ≥ 1	0.889	0.390–2.025	0.779
No involvement of DBS vs. involvement of DBS	0.756	0.386–1.479	0.413
Refractory vs. relapse	2.152	0.909–5.094	0.081
Asymptomatic vs. symptomatic	0.426	0.189–0.960	**0.040**
Time in remission < vs. ≥ 12 months	0.773	0.313–1.906	0.576
Any other treatment vs. WBRT/focal radiotherapy	2.239	0.450–11.139	0.325
Any other treatment vs. HD-MTX-based therapy	3.720	0.548–25.275	0.179
Any other treatment vs. TMZ monotherapy/TMZ + rituximab	1.269	0.253–6.372	0.772
Best supportive care vs. any salvage treatment	6.018	0.968–37.406	0.054

*Abbreviations:* DBS = deep brain structures, HD-MTX = high dose methotrexate, HR = hazard ratio, ICV = intracerebroventricular, KPS = Karnofsky performance score, LDH = lactate dehydrogenase, TMZ = temozolomide, WBRT = whole brain radiotherapy

### Long-term survival

Eight patients (13.1%) survived ≥ 3 years after diagnosis of cerebral relapse or progression of PCNSL. Three patients were still alive at censoring date, three had eventually died due to progression and two for unknown reasons. Salvage treatments in those eight patients at first relapse were TMZ in three, WBRT in three, HD-MTX monotherapy followed by WBRT in one patient, and rituximab, HD-MTX and TMZ (2.5 years after last day of prior treatment) in the remaining patient. Response to treatment of first relapse was CR/CRu in all eight. Median age in this patient group was 71.5 years (range 26–81 years) and median KPS at diagnosis of relapse or progression was 55 (range 40–90). Median KPS after completion of treatment was 70 (range 50–90). Two patients were able to live independently at home following first salvage treatment, four patients lived at home supported by family or nursing service, and two patients lived in a care facility.

### Treatment duration, hospitalization time and clinical status after treatment

Median duration of treatment for all patients, who received salvage treatment (and not BSC) was 40 days (range 14–270 days). Median hospitalization time was 20 days (range 0–99 days) in the entire patient cohort and 26 days (range 0–99 days) in patients, who received any salvage treatment.

Nine patients, who received BSC only, and four patients, who died treatment related, were not assessable for evaluation of KPS after treatment. In the remaining 41 assessable patients, median KPS at first relapse of PCNSL before the beginning of salvage treatment was 60 (range 30–100). Median KPS after treatment termination or completion was 50 (range 20–90). For the 16 patients with CR/CRu or PR after treatment median KPS improved from 60 (range 40–90) before to 70 (range 50–90) after treatment. In 25 patients with PD during salvage treatment the median KPS before and after treatment decreased from 60 (range 30–100) to 40 (range 20–70).

Discharge destination after completion of treatment in those 41 patients were as follows: Five patients (12.2%) were able to live independently at home after treatment. In 25 patients (60.9%) living at home was possible with support of family and/or nursing service. Four patients (9.8%) were transferred to a care facility. One patient was admitted to a hospice (2.4%). Six patients (14.6%) were hospitalized until next progression/relapse.

Details on response to treatment, survival, treatment duration, hospitalization time and clinical status before and after treatment divided by treatment groups are presented in Table 3.

**Table 3:** Clinical characteristics, response to treatment, survival, treatment duration and hospitalization time after first relapse/progression divided by treatment groups (n = 54)

Treatment group	No. of patients	Median age	Response to treatment	Median OS (months)	Median treatment duration (days)	Median hospitalization time (days)	Median KPS at relapse/ progression	Median KPS after treatment^d^
TMZ-based ^a^ (n = 15 TMZ monotherapy, n = 6 TMZ + rituximab)	21	73	CR/CRu n = 4 PR n = 1 PD n = 13 Early death n = 3	4.8	38	15	60	50
HD-MTX-based ^b c^ (n = 8 HD-MTX + TMZ, n = 2 HD-MTX monotherapy, n = 1 HD-MTX + ifosfamide)	11	72	CR/CRu n = 5 PR n = 1 PD n = 4 Early death n = 1	5.8	97	55	60	60
WBRT/ focal radiotherapy	11	72	CR/CRu n = 5 PR n = 1 PD n = 5	7.5	40	13	60	50
Other treatment at cerebral relapse (n = 1 temsirolimus [24], n = 1 rituximab monotherapy)	2	74	PD n = 2	2.1/7.0	27	27	60	40
Best supportive care	9	77	Not applicable	0.8	0	7	50	-

^a^ 15 of 21 patients were able to be discharged from the hospital and receive treatment at home

^b^ one of these patients received WBRT after infectious complications and only PR after four cycles of HD-MTX monotherapy and one patient received consolidating local radiotherapy

^c^ median time from last day of prior treatment to diagnosis of relapse was 22.4 months (range 3.8–83.5 months) in this group

^d^ patients with treatment related death were not assessable for this analysis

Abbreviations: CR = complete remission, HD-MTX = high dose methotrexate, KPS = Karnofsky performance score, PD = progressive disease, PR = partial remission, RT = radiotherapy, TMZ = temozolomide, WBRT = whole brain radiotherapy

### Treatment at further relapses

Thirty-one second relapses were observed in the entire cohort of 54 patients. At cerebral second relapse 12 patients received best supportive care, eight patients received WBRT, one received HD-MTX + TMZ, one received TMZ + rituximab. Two patients were treated with temsirolimus within a clinical trial [24], one patient received topotecan and one patient rituximab + thiotepa + cytarabine. One systemic second relapse was observed and treated with rituximab, cyclophosphamide, doxorubicine, vincristine and prednisolone (R-CHOP). Three ocular relapses were observed and treated with trofosfamide (n = 1) and ocular radiotherapy (n = 1). One patient with ocular relapse died of pneumonia associated sepsis before initiation of ocular radiotherapy. At third relapse nine patients received palliative treatment and one patient was treated with TMZ.

## Discussion

In this retrospective study, we analyzed survival and prognostic factors in 54 patients with PCNSL refractory to first line treatment or at first cerebral relapse of PCNSL and not eligible for HDC-ASCT, who received heterogenous salvage treatments. In patients with an unfavorable prognosis and with virtually no chance of cure questions arise on aspects beyond survival. Based on clinical experience concerns relate to treatment duration, required hospitalization time and most importantly to the likelihood of significant clinical improvement with therapy, ideally with the aim to live independently.

The most frequently used salvage treatments for cerebral relapse or refractory disease at our hospital between 2006–2022 were temozolomide (+ rituximab), HD-MTX-rechallenge and WBRT. Median OS in this series, that was restricted to patients not eligible for HDC-ASCT was low with 4.8 months. OS of patients treated with TMZ, which was 4.8 months in our study, was comparable to results reported in a previous study [15]. For patients, who were treated with HD-MTX-rechallenge, OS was inferior as compared to results of other series (5.8 months in our study vs. 41 months [10] and 61.9 months [11] in previous series). Only one patient in our study, who had received HD-MTX rechallenge at cerebral relapse without WBRT or other subsequential treatments survived for ≥ 3 years. Both studies on HD-MTX rechallenge [10,11] had included a small number of patients, that had received HDC-ASCT as first line treatment, pointing to the fact that those patients at first line were young and fit enough to receive an aggressive treatment initially, while in the present series more vulnerable and fragile patients had been included. Furthermore, in one of the studies four of 39 patients had received HDC-ASCT as consolidation treatment after HD-MTX rechallenge [10]. It is noteworthy, that in the present series a consecutive group of patients has been analyzed, selected by virtue of poor prognosticators only. These aspects may partly explain the superior results with HD-MTX rechallenge in these studies compared to the results of our analysis, however, the difference remains substantial.

The application of salvage treatment itself compared to BSC was a positive prognostic factor in univariate analysis in our study as previously reported by other groups [2, 27]. Apart from application of any salvage treatment (vs. BSC), positive prognosticators in our study were asymptomatic relapse and younger age. In a series on 256 patients with r/r PCNSL, asymptomatic relapse had previously been shown to be positively prognostic compared to symptomatic relapse [2]. This might be due to an earlier detection of progression or relapse and therefore to lower lymphoma burden. Regarding the influence of age on prognosis, an age cut-off at 75 years was chosen in our analysis, because HDC-ASCT is usually considered in selected patients up to 75 years of age. Only four of the 26 patients in our cohort, who were 75 years or older, survived for more than 12 months after diagnosis of progression or relapse. These four patients had received different salvage treatments (n = 2 WBRT, n = 1 TMZ, n = 1 HD-MTX + TMZ). Considering these results, BSC seems to be a justifiable option in some patients ≥ 75 years and a short hospitalization by itself is a therapeutic aim.

Patients treated with TMZ (+ rituximab) or radiotherapy had a relatively short median hospitalization time of 15 days and 13 days as compared to patients, who received HD-MTX-based therapy as salvage treatment (55 days). In addition, patients with HD-MTX-based treatment spent a larger part of the whole treatment duration hospitalized when compared to those treated with TMZ (+ rituximab) or radiotherapy. TMZ has been used as a single agent or in combination with rituximab in 21 patients in this series. Its availability as an oral formulation is associated with applicability on an outpatient basis (as was the case in 15 TMZ patients of this series). Almost all r/r PCNSL patients eventually relapse and many already show PD during treatment with temozolomide (+ rituximab) salvage and median PFS/OS reported in previous studies were only 2.8/3.9 months [15] and 1.9 months /not reached [28]. However, in our series long term survival of three or more years has been observed in three of 21 patients. Applicability on an outpatient basis, a low rate of side effects, absence of neurotoxicity and a possibility of a long-lasting therapeutic response, albeit small, in our view justifies an attempt of TMZ-based treatment at first relapse/progression in patients, who are not eligible for HDC-ASCT and who do not qualify for prospective clinical studies. The long hospital stays for HD-MTX rechallenge should be considered and weighed thoroughly against the likelihood of successful treatment. Previously described positive prognostic factors for HD-MTX rechallenge were KPS [10] and (long) time to first relapse [11]. In the present series neither KPS nor time to first relapse were prognostic in patients with cerebral relapse or progression, however, this might be due to the small patient population. For WBRT the median hospitalization time (13 days) was low in this series, however, the risk of delayed neurotoxicity after WBRT at relapse is to be carefully considered [12, 13] and argues against WBRT at relapse, particularly in elderly patients. Neuropsychological follow up was not performed routinely in our patient cohort, however, one patient had severe dementia subsequently to WBRT at first relapse and died from pneumonia while being bedridden.

Median KPS in assessable patients (n = 41) was 60 before treatment and 50 after treatment. Not surprisingly, patients with response to treatment had an improved KPS after treatment, while patients with PD had a lower KPS. However, even patients with response to therapy had a median KPS of only 70 after treatment, which likely reflects the overall reduced clinical state of our patient cohort, independent of symptoms caused by lymphoma. In patients treated with TMZ (+ rituximab) median KPS at diagnosis of relapse or progression was 60 compared to 50 after treatment. This is at least partly explained by the relatively high number of patients (n = 13) with PD during treatment in this group.

Our study is limited by its retrospective design and by heterogenous salvage treatments. The rather small patient population treated in a single tertiary care center limits the statistical power of the analysis and the generalizability of the results. We did not have detailed information on activities of daily living, quality of live and treatment toxicity. Further, no standardized neurocognitive and neuropsychological assessment at diagnosis or during follow-up was available for this retrospective analysis. As the time period covered is 2006–2022, a bias induced by changing clinical practice over the last years is possible, e.g., an increasing number of elderly patients is considered eligible for HDC-ASCT.

## Conclusions

In conclusion, survival of patients with r/r PCNSL not eligible for HDC-ASCT remains poor and none of the currently available treatment options is curative. The combined treatment goal of prolonging survival with a minimum of side effects and the possibility of treatment on an outpatient basis argue in favor of temozolomide treatment. Novel treatment strategies are urgently needed and are currently evaluated in clinical trials. Future treatment perspectives comprise a wide range of targeted therapies. Possible targets are in particular the B-cell receptor and the mammalian target of rapamycin pathways [20. 24]. Immunmodulatory drugs like lenalidomide an pomalidomide have also shown potential for targeted treatment of r/r PCNSL [21–23].

As an overexpression of the programmed cell death protein 1 (PD1) receptor and the PD1 ligand is frequently found in PCNSL [29], the use of immune checkpoint inhibitors is also a promising approach. However, only very small series have been published so far [30, 31]. The results of a larger prospective trial on the PD1 inhibitor nivolumab (NCT 02857426) are pending. CD 19-directed chimeric antigen receptor (CAR) T cell therapy, which has shown remarkably good results in patients with relapsed or refractory systemic diffuse large B cell lymphomas (DLBCL) [32], is another promising treatment option in r/r PCNSL. Due to the potential CNS toxicity caused by this treatment, patients with PCNSL or CNS involvement of systemic DLBCL had been excluded from the initial trials on CD 19 directed CAR T cell therapy for systemic DLBCL [32]. More recently, results of a small prospective series and a systematic review have demonstrated responses in a relevant fraction of r/r PCNSL patients treated with CD 19 directed CAR T cells [33, 34]. Several prospective trials evaluating CAR T cell treatment in PCNSL are currently recruiting (e.g. NCT 04608487, NCT 04443829).

## Data Availability

The data that support the findings of this study are available from the corresponding author upon reasonable request.

## Abbreviations

BSC: Best supportive care
CAR-T: Chimeric antigen receptor T (CAR-T)
CCI: Charlson comorbidity index
cMRI: Cerebral magnet resonance imaging
CR: Complete remission
CRu: Complete remission unconfirmed
CT: Computer tomography
CSF: Cerebrospinal fluid
FDG: Fluorodeoxyglucose
HDC-ASCT: High dose chemotherapy with autologous stem cell transplantation
HD-MTX: High dose methotrexate
KPS: Karnofsky performance ccore
mTOR: Mammalian target of rapamycin
OS: Overall survival
PCNSL: Primary central nervous system lymphoma
PFS: Progression free survival
PET: Positron emission tomography
PD: Progressive disease
PR: Partial response
R-CHOP: Rituximab, cyclophosphamide, doxorubicine, vincristine and prednisolone
r/r PCNSL: Relapsed or refractory primary CNS lymphoma
TMZ: Temozolomide
WBRT: Whole brain radiotherapy
